# High occurrence of food insecurity in young people attending a youth mental health service in regional Australia

**DOI:** 10.1111/1747-0080.12755

**Published:** 2022-07-07

**Authors:** Katherine Kent, Sandra Murray, Denis Visentin, Tamieka Mawer, Courtney J. McGowan, Andrew D. Williams, Sibella Hardcastle, Heather Bridgman

**Affiliations:** ^1^ School of Health Sciences Western Sydney University Sydney New South Wales Australia; ^2^ College of Health and Medicine University of Tasmania Launceston Tasmania Australia

**Keywords:** adolescent, food security, mental health, young adult

## Abstract

**Aim:**

Despite the relationship between food insecurity and poor mental health, food insecurity in young people attending mental health services in Australia remains understudied. This study aimed to determine the occurrence and predictors of food insecurity, and the relationship with dietary factors in young people attending a mental health service.

**Methods:**

A cross‐sectional online survey was conducted in a sample of young people (15–25 years) who attended a mental health service in Launceston, Australia. The survey utilised a single‐item food insecurity screening tool and eight demographic, health and service use questions. Five questions determined self‐reported intake of fruit, vegetables, breakfast, water, sugar‐sweetened beverages and takeaway foods. Binary logistic regression determined predictors of food insecurity. Cross‐tabulations determined differences in dietary intake according to food security.

**Results:**

Of survey respondents (*n* = 48; 68% female), 40% (*n* = 19) were food insecure. Respondents living out of home or in unstable accommodation were at significantly higher risk of food insecurity (odds ratio [OR]: 4.43; SE: 0.696; 95% CI: 1.13–17.34; *p* = 0.032) compared to those living with their parents. Those receiving government financial assistance (OR: 5.00; SE: 0.676; 95% CI: 1.33–18.81; *p* = 0.017) were also at significantly higher risk of food insecurity. Regardless of food security status, self‐reported intake of fruits, vegetables and breakfast were low, and respondents regularly consumed takeaway foods and sugar‐sweetened beverages.

**Conclusions:**

There was a high occurrence of food insecurity and poor dietary intake in young people attending a youth mental health service demonstrating that initiatives to support access to healthy food in this group should be a priority, with potential benefits for mental health outcomes.

## 1 INTRODUCTION

Food security exists ‘when all people, at all times, have physical, social and economic access to sufficient, safe and nutritious food that meets their dietary needs and food preferences for an active and healthy life’.^1^ Food insecurity is the absence of these factors, and it persists even in high‐income countries including Australia. The prevalence of food insecurity in a representative sample of the Australian population has been conservatively estimated to be approximately 5%^2^ but is reported to be much higher in socioeconomically disadvantaged groups.^3^ A review of Australian studies in various population groups has reported that food insecurity prevalence statistics varied greatly depending on the measurement tool applied and subgroup studied, with the highest prevalence observed in very remote communities.^4^

Overall, there is a paucity of research about food security in adolescents and young adults in Australia, with a focus on families with young children^5^ and older adults.^6^ Some limited data regarding food insecurity in specific sub‐groups of vulnerable young people in Australia exists with those experiencing homelessness or living in supported accommodation, experiencing a substantially higher occurrence of food insecurity (66% food insecure) compared to the wider adult population.^7^ However, there are a lack of food security data for young people in general in Australia and an absence of literature which links food insecurity with health outcomes in this group, despite the growing body of convincing evidence about the relationship between food insecurity and poor mental^8^ and physical health outcomes^9^ in other populations. In international literature within other high‐income countries, food insecurity is shown to be higher in young people when compared to older age groups. For example, a recent analysis in Canada reported that 30% of young people (aged 16–30 years) were living in food‐insecure households, and these food‐insecure respondents were two and a half times more likely to experience poor mental health outcomes.^10^ Another study conducted in the United States reported that more than 23% of young people were food insecure, which was associated with poorer diet quality and increased prevalence of substance use.^11^ Furthermore, food insecurity was associated with increased mental health care service utilisation in both younger and older adults.^12^

Young people who are food insecure are more likely to have poorer diets.^13^ The nutritional consequences of food insecurity, such as eating less food or lower quality food, are critical for young people as adequate nutrition is required for optimal growth and development both physically and mentally.^14^ In addition, adolescence and young adulthood are key windows of development as during this period, eating behaviours are established and many psychiatric conditions emerge for the first time.^15^ In Australia, 14% of young people have been diagnosed with a mental disorder.^16^ A growing body of international evidence highlights the importance of healthy nutrition behaviours for supporting positive mental health outcomes in young people.^17^ Unhealthy dietary patterns are consistently linked to poorer mental health outcomes in young people.^18^ Furthermore, as food insecurity increases, the odds of young people having a mental disorder also increase.^19^ This is supported by evidence from a recent review which evaluated the psychosocial outcomes related to food insecurity for young people, reporting that food insecurity is associated with depression, anxiety, suicidal ideation, substance use disorder and an increased need for psychological care.^20^ Therefore, increasing our understanding of the role of diet and food insecurity in young people with poor mental health should be a priority.

Although a great deal of research exists on the burden of food insecurity in many vulnerable groups within Australia,^4^ studies in young people attending mental health services are scarce. A critical window exists for screening young adults in this setting to prioritise interventions for both their future mental health and food security. However, to the authors' knowledge, no research on the occurrence and demographic predictors of food insecurity nor its relationship with dietary intake has been conducted within this group. This is a major research gap as mental disorders are prevalent conditions among young Australians. Importantly, gathering this evidence could provide additional support in regard to the role dietetics services may have in the prevention and early intervention of mental illness.^16^ Therefore, this cross‐sectional study in a sample of young people (aged 15–25 years) attending a youth mental health service in regional Australia aimed to answer the following research questions:

- What is the occurrence of food insecurity in young people attending a mental health service?
- What are the demographic predictors of food insecurity in this group?
- What is the relationship between food insecurity and measures of dietary intake such as self‐reported intake of fruits, vegetables, breakfast, takeaway foods and sugar‐sweetened beverages?

The main hypothesis of this research was that there will be a high burden of food insecurity within this population and that discrete demographic groups of young people attending a youth mental health service will experience food insecurity. Secondly, we hypothesised that there will be a relationship between food security status and poor dietary habits, namely lower intakes of fruits, vegetables as well as the consumption of breakfast, and/or higher intakes of takeaway and sugar‐sweetened beverages.

## 2 METHODS

The *headspace* Launceston (Cornerstone Youth Services) is a youth mental health service based in the regional city of Launceston in Tasmania, an island state of Australia sitting south of the mainland. Tasmania has a population size of nearly 542 000 people^21^ with a median age of 42 years. In 2016,^22^ there were approximately 91 000 people aged 10–24 years, comprising around 18% of the Tasmanian population. Tasmania is highly diverse in terms of remoteness, incorporating the capital city of Hobart (regional city), and other major regional centres including Launceston, Burnie and Devonport, as well as many rural communities.^23^

There are several services in Tasmania providing support, counselling, and therapeutic interventions for young people experiencing poor mental health.^24^ Specifically, *headspace* Launceston provides early intervention mental health and wellbeing services to young people aged 12–25 years including counselling, psychology and mental health services delivered by a range of practitioners including mental health workers, general practitioners and psychiatrists. Clients are referred to external dietetic support upon request from clients, with limited funding to support this service internally for their clients.

This study is a sub‐analysis of a larger project,^25^ which aimed to inform improvements in service delivery to support young people accessing *headspace* Launceston to improve nutrition and physical activity behaviours in their clients. Ethical approval to conduct this study was obtained from the Tasmania Social Science Human Research Ethics Committee (H0023475) and the study was conducted in accordance with the Declaration of Helsinki. Compliance to the STrengthening the Reporting of OBservational studies in Epidemiology – Nutritional Epidemiology (STROBE‐nut) tool has been addressed.

A quantitative, online survey was developed by the project team for the purpose of this study. Only the relevant questions for the analyses underpinning this manuscript are described. For a full list of the survey outcomes please see the published report.^25^ The survey was assessed for face validity and legibility by several stakeholders including *headspace* management and staff, members of headspace Launceston's Youth Reference Group (who are made up of young people passionate about mental health and wellbeing), and a high school teacher. The survey was also edited using Grammarly (2021 © Grammarly Inc) to ensure the reading level for the survey was no higher than the age of 12 years, to suit the known literacy level in the area.

The survey measured food security status using a single item food security screening tool, which is the most commonly applied tool in Australia, including in national health surveys.^4^, ^26^ The question asks “Over the past year, have you ever run out of food and couldn't afford to buy any more?”. An affirmative response to this question indicates food insecurity. This tool was selected to minimise participant burden compared to other food security assessment tools.

Demographic characteristics were collected including age (continuous variable), gender (male, female, self‐described), living situation (living with parents, living independently, living in short term/unstable accommodation or homeless), postcode and town name, whether they were of Aboriginal and/or Torres Strait Islander background (yes or no), whether they were receiving government financial assistance (yes or no) and the number of visits to *headspace* over the past 12 months (continuous).

Brief nutrition‐related questions were based on recommendations made in a review of short questions to assess the food consumption of children in Australia.^27^ Two questions determined how many serves of fruits and vegetable participants usually ate each day (including fresh, frozen, and tinned varieties). The self‐reported daily frequency of consuming fruit was recorded as: either do not consume, 1 serve, 2 serves or more than 2 serves. For vegetables, response options were recorded as: do not consume, 1 serve or less, 2 serves, 3 serves, 4 serves, 5 serves, or more than 5 serves. Four questions determined how frequently participants usually ate breakfast, drank water, sugar‐sweetened beverages, and ate take‐away food, with the response options including never/rarely, less than once a week, weekly, a couple of days a week, daily, more than once per day.^27^ Portion size guides were provided to participants to improve the estimation of their self‐reported intake of these foods.

Participants were young people aged between 15 and 25 years who self‐identified as having contact with *headspace* Launceston within the past 12 months. Young people aged 12–14 years and attending *headspace* were not eligible due to ethical concerns about providing assent. Data collection was undertaken between February and June 2021 through LimeSurvey (LimeSurvey GmbH, Hamburg, Germany). Participants were recruited through *headspace*, where clinicians distributed printed flyers promoting the study to clients attending the service. The flyers had a link and QR code to enter the survey. Due to the low uptake via this method, a 5‐week paid advertisement was run on social media platforms Facebook and Instagram with the parameters set to target 15–25 year olds living in Launceston or surrounds (within a 50 km radius). Over this period, the survey post reached 26 253 young people, with 621 survey link clicks that led to the information sheet that specified eligibility, and the opportunity to review the screening questions to enter the survey. Of this, there were 140 attempts at the screening questions that were employed to determine informed assent (those aged 15–17 years) and consent (those aged 18–25). Participants had to correctly confirm that (i) they were not under a child protection order; (ii) that no one would know they have done the survey; (iii) and, that they understood if they started the survey, they could stop at any time. Of the 140 attempts, 53 completed and answered all screening questions correctly. There were several instances of duplicate IP addresses being used within a matter of minutes with a high level of homogeneity between survey answers, indicating a lack of authenticity in these survey responses. Therefore, duplicate surveys received within 10 min from the same IP addresses were deleted, leaving a final sample size of 48.

Data were exported from the online software into Microsoft Excel (Microsoft Corporation, 2018), then transferred into IBM SPSS Statistics for Windows, version 26.0 (IBM Corp., Armonk, NY, USA) for analysis. All available survey data were used in the analyses, with surveys excluded if a response to the food security question were not provided. If data were missing for other demographic characteristics, they were only excluded for the relevant univariate analyses or cross‐tabulations to retain as much data as possible. The significance level for all analyses was set at *p* < 0.05.

Food security status was determined by classifying an affirmative response to the single item question as food insecure and a negative response as food secure. To assist in comparative analyses and due to the distribution of the data, binary variables were generated, with age coded into those aged 15–17 years or 18–25 years, and the number of visits to *headspace* were coded into 1–3 visits or 4 or more visits using a data‐driven approach. Living situation options ‘living in short term/unstable accommodation’ and ‘homeless’ were merged into one group due to low cell counts. Postcodes and town names were used to classify people according to levels of rurality using the Modified Monash Model^28^ (levels 1–6), which were then collapsed into a binary variable of regional^2^ and rural.^3^, ^4^, ^5^, ^6^

Intake of fruits and vegetables was interpreted against the Australian Guide to Healthy^29^ Eating which recommends adolescents consume at least 2 serves of fruit and 5 serves of vegetables per day. Therefore, participants were coded into binary groups (‘meeting recommendations’, ‘not meeting recommendations’). However, due to low cell counts in the meeting recommendations group, the fruit and vegetable groups were further recoded into ‘eaten daily’ or ‘not eaten daily’. In addition, for breakfast, a binary variable was coded by collapsing for breakfast (‘eaten daily’ or ‘not eaten daily’); water (‘multiple times per day’; ‘only once a day or less’); sugar‐sweetened beverages and takeaway food (‘weekly or more’; ‘less than once a week’).

Cross‐tabulations with chi‐square statistics were used to compare the proportions of food secure and food‐insecure respondents, according to their demographic characteristics, and to compare dietary intake variables by food security status. Binary logistic univariate regression was used to determine the demographic predictors of food security status in the sample.

## 3 RESULTS

Survey respondents providing a response to the food security screening question (*n* = 48) were predominantly classified as food secure (60%; *n* = 29) and 40% (*n* = 19) were classified as food insecure (Table 1). Of the study sample, most were aged 18–25 years (65%), female (68%), living in an outer regional area (89%), not of Aboriginal and/or Torres Strait Islander background (89%) and had visited *headspace* more than three times over the past 12 months (Table 1). Nearly half of the study sample (49%) were living independently out of the family home, and 44% were living with their parent/s. Less than a quarter (21%) of those living with their family were food insecure compared to nearly half (48%) who were living independently. A minority of the study sample (7%) were either ‘homeless’ or ‘living in unstable accommodation’ and all of these respondents were food insecure (Table 1). More than a third (36%) of survey respondents were receiving government financial assistance. However, the majority of those receiving these payments were food insecure (63%) (Table 1). A greater proportion of those who attended *headspace* 4 or more times in the past 12 months were food insecure (46%) compared to those who attended 3 times or less (30%) (Table 1).

**TABLE 1:** Demographic characteristics of the study sample of young people attending a youth mental health service according to food security status showing the proportion of the total study sample in each category (total column); and the proportion (*n* [%]) of food secure and food‐insecure respondents in each category (food‐secure and food‐insecure columns)

Characteristic	Category	Total *n* (%)	Food secure *n* (%)	Food insecure n (%)	χ^2^	*p*‐Value
Age (*n* = 43)	15–17	15 (34.9)	10 (66.7)	5 (33.35)	0.371	0.543
	18–25	28 (65.1)	16 (57.1)	12 (42.9)		
Gender (*n* = 44)	Female	30 (68.2)	19 (63.3)	11 (36.7)	0.205	0.903
	Male	12 (27.3)	7 (58.3)	5 (41.7)		
	Self‐identity as other	2 (4.5)	1 (50.0)	1 (50.0)		
Living situation (*n* = 43)	At home with parent/s	19 (44.2)	15 (78.9)	4 (21.1)	7.877	0.019
	Living out of home	21 (48.8)	11 (52.4)	10 (47.6)		
	Unstable accommodation/homeless	3 (7.0)	0 (0.0)	3 (100.0)		
Rural classification (modified Monash model^28^) (*n* = 41)	1–2	36 (87.8)	22 (61.1)	14 (38.9)	0.002	0.962
	3–6	5 (12.2)	3 (60.0)	2 (40.0)		
Receiving government financial assistance (*n* = 44)	No	28 (63.6)	21 (75.0)	7 (25.0)	6.039	0.014
	Yes	16 (36.4)	6 (37.5)	10 (62.5)		
I am Aboriginal and/or Torres Strait Islander (*n* = 44)	No	39 (88.6)	24 (61.5)	15 (38.5)	0.004	0.947
	Yes	5 (11.4)	3 (60.0)	2 (40.0)		
Number of visits to *headspace* (*n* = 42)	1–3	20 (47.6)	14 (70.0)	6 (30.0)	1.061	0.303
	4+	22 (52.4)	12 (54.5)	10 (45.5)		
Received a mental health diagnosis (*n* = 44)	No	18 (40.9)	13 (48.1)	5 (29.4)	1.515	0.218
	Yes	26 (59.1)	14 (51.9)	12 (70.6)		
Total		48	29 (60.4)	19 (39.6)		

The univariate regression (Table 2) identified that respondents who were living out of home and in unstable accommodation were four times more likely to be food insecure compared to those living at home. Furthermore, those receiving government financial assistance were five times more likely to be food insecure compared to those not receiving these payments (Table 2).

**TABLE 2:** Univariate regression demonstrating the relationship between demographic characteristics and risk of food insecurity in a sample of young people attending a youth mental health service (*n* = 48)

		Odds ratio	SE	Confidence intervals	*p*‐Value
Age, years (*n* = 43)	<18	—	—	—	—
	≥18	1.500	0.668	0.405–5.552	0.544
Gender (*n* = 43)	Female	—	—	—	—
	Male	1.234	0.697	0.314–4.840	0.763
	Self‐identity as other	1.727	1.464	0.098–30.450	0.709
Living situation (*n* = 43)	At home with parent/s	—	—	—	—
	Living out of home/ Unstable accommodation/ Homeless	4.432	0.696	1.133–17.341	0.032
Rural Classification—Modified Monash Model^28^ (*n* = 41)	1–2	—	—	—	—
	3–6	1.048	0.975	0.155–7.079	0.962
Receiving government financial assistance (*n* = 44)	No	—	—	—	—
	Yes	5.000	0.676	1.329–18.814	0.017
I am Aboriginal and/or Torres Strait Islander (*n* = 44)	No	—	—	—	—
	Yes	1.067	0.971	0.159–7.145	0.947
Number of visits to *headspace* (*n* = 42)	1–3	—	—	—	—
	≥4	1.944	0.649	0.545–6.940	0.306
Received a mental health diagnosis (*n* = 44)	No	—	—	—	—
	Yes	2.229	0.657	0.615–8.078	0.223

Most respondents (70%) consumed fruit daily and around half (52%) reported consuming vegetables daily (Table 3). However, only 18% and 8% of survey respondents met the public health recommendations for the consumption of fruits and vegetables intake, respectively. In addition, only 33% of survey respondents consumed breakfast daily (Table 3). However, there was no significant difference between food secure and food‐insecure respondents for these dietary intake variables. Most respondents (63%) consumed water more than once per day, but this result was being driven by water consumption in food secure respondents (76%) but not food‐insecure respondents (42%). There was no difference between food secure and food‐insecure respondents' consumption of takeaway or sugar‐sweetened beverages, and overall, most survey respondents consumed these more than once per week (58% and 54%, respectively; Table 3).

**TABLE 3:** Dietary intake according to food security status in the study sample of young people attending a youth mental health service (*n* = 48) showing the proportion of the total study sample in each category of dietary intake (total column); and the proportion (*n* (%)) of food secure and food‐insecure respondents in each category of dietary intake (food‐secure and food‐insecure columns)

		Total *n* (%)	Food secure *n* (%)	Food insecure *n* (%)	χ^2^	*p*‐value
Fruit intake	Not eating daily	14 (29.2)	7 (24.1)	7 (36.8)	0.897	0.344
	Eating daily	34 (70.8)	22 (75.9)	12 (63.2)		
Vegetable intake	Not eating daily	23 (47.9)	12 (41.4)	11 (57.9)	1.255	0.263
	Eating daily	25 (52.1)	17 (58.6)	8 (42.1)		
Breakfast intake	Not eating daily	32 (66.7)	17 (53.1)	15 (46.9)	2.134	0.144
	Eating daily	16 (33.3)	12 (75.0)	4 (25.0)		
Water intake	Multiple times per day	18 (37.5)	7 (24.1)	11 (57.9)	5.851	0.018
	Once a day or less	30 (62.5)	22 (75.9)	8 (42.1)		
Sugar sweetened beverages intake	Less than once a week	20 (41.7)	11 (37.9)	9 (47.4)	0.421	0.517
	Weekly or more	28 (58.3)	18 (62.1)	10 (52.6)		
Takeaway food intake	Less than once a week	12 (25.0)	6 (20.7)	6 (31.6)	0.726	0.394
	Weekly or more	36 (75.0)	23 (79.3)	13 (68.4)		

## 4 DISCUSSION

This study is the first in Australia to screen for the occurrence, and determine the demographic predictors, of food insecurity in a convenience sample of young people attending a youth mental health service. The study also assessed the relationship between food security status and dietary intake in this group. The results suggest that the occurrence of food insecurity in this vulnerable group is much higher than the wider Australian population, and that young people living out of home or living in unstable accommodation, and those receiving government financial assistance, are at the highest risk of food insecurity. The results are not surprising, given the relationship between food security status and social disadvantage and low incomes that persists across all age groups in Australia, but particularly in young people.^3^, ^30^ The results point to a clear opportunity to implement regular screening for food insecurity for young people attending a youth mental health service who would benefit from targeted support enabling them to access sufficient, safe and healthy food. Given the relationship between mental health, diet quality and food security,^8^ this could improve mental and physical health outcomes in this vulnerable group.

In the current study, 40% of survey respondents were food insecure, which is very similar to the 38% of university students experiencing food insecurity in the same state of Tasmania, Australia,^31^ and slightly lower than the prevalence in a sample of Australian adults with mental illness (45%).^32^ In comparison, these statistics are substantially lower than those included in a report from Foodbank (an Australian emergency food relief agency) who reported that in July 2021, in a representative sample of Australian adults, 65% of people aged 18–25 years were food insecure, which was higher than any other age group and most likely related to the loss of casual work during the COVID‐19 pandemic in Australia.^33^

Critically, the current study found that young people living independently or in unstable accommodation, and those receiving government financial assistance were at substantially higher risk of food insecurity. It is well established that people or households with low economic resources are at high risk of food insecurity, and that the primary reason for food insecurity in Australia is material hardship and inadequate financial resources.^34^ Young people in Australia also face substantial financial challenges due to precarious casual work arrangements^35^ and unaffordable housing.^36^ Indeed, in a study of young people living in community housing, private rentals, or staying with friends and family, the occurrence of food insecurity was 85%.^7^ Until adequate government financial assistance is provided, food insecurity will continue to be pervasive in its recipients, regardless of age, given the government assistance payments fall several hundred dollars a week below the poverty line.^37^ The results of a national Think Tank to address the mental health crisis in young Australians have been published, calling on the Australian government to urgently raise the rate of government financial assistance payments as a measure to reduce the growing issue of poor mental health in young people, and reduce the burden on over‐stretched mental health services.^38^ Young people with mental illness may be at substantially higher risk of long‐term reliance on government financial assistance due to their limited capacity to work when symptoms of mental illness are distressing them.^39^

The current study indicated that a higher proportion of respondents who were food insecure attended the mental health service more frequently and had received a mental health diagnosis compared to those who were food secure, but this was not significantly different between groups. This is most likely due to the small study sample resulting in underpowered subgroup analyses. In international research, food insecurity has been associated with higher mental health service use,^12^ and this factor should be a consideration for future research in larger sample sizes. Further understanding of the relationship between mental health service use and food security in young people may provide further support for dietetic involvement in multidisciplinary healthcare teams. For people regularly attending mental health services, it has been suggested that dietitians could provide integral support for improving food security and dietary habits through nutritional assessments, interventions, documentation of care, patient education and staff training,^40^ with benefits for both physical and mental health outcomes.^41^, ^42^

In the current study, food security was not related to lower dietary intakes of fruit, vegetables or breakfast. However, most young people in our study did not meet the Australian Government's dietary recommendations for these foods regardless of their food security status. In 2017–2018, it was reported that of young Australians aged 15–24 years, 18% did not consume a whole serving of fruit daily, and 9% did not consume a serving of vegetables daily.^43^ In the current study, dietary intake of fruits and vegetables was substantially worse, with nearly half of respondents (48%) not eating vegetables daily and 30% of respondents not eating fruit daily. This highlights the extent of poor diet within this vulnerable sample, and is similar to dietary outcomes in a vulnerable group of young adults experiencing homelessness.^7^ Furthermore, in Australian adults with severe mental illness, food‐insecure respondents were significantly less likely to consume fruit, vegetables and protein‐based foods at least daily.^32^ Another Australian study determined that young people diagnosed with a severe mental illness had low overall diet quality.^44^ In that particular study, 43% of energy was derived from discretionary (energy dense, non‐nutritious) foods.^44^ The study also reported that poor dietary intake was observed even in the early stages of mental illness, which likely contributed to poor physical and mental health outcomes in that group.^44^

Most participants in the current study, regardless of food security status, reported consuming sugar‐sweetened beverages and takeaway foods at least once a week, indicating that these are potentially contributing substantially to overall dietary patterns. In addition, the frequency of consumption of water as a beverage was higher in food secure young people in our study. This may relate to the relatively better dietary habits or better access to fresh water in this group. Lastly, in the current study, only a third of respondents reported consuming breakfast daily regardless of food security status, which is lower than in other studies of young people in Australia showing 92% consume breakfast daily.^45^ However, a study by Pepetone et al.^46^ reported that young women in food‐insecure households reported preparing a lower proportion of breakfasts at home, which was related to financial precarity, rather than lack of food skills. These findings suggest a need for interventions to substantially improve the dietary habits of young people attending youth mental health services, regardless of food security status. Future research should prioritise determining the specific barriers and enablers towards achieving high diet quality in this group.

While this study presents some of the first results of food insecurity in young people attending a mental health service, the study should be considered in the context of some limitations. First, as is common for this cohort of vulnerable young people, the study has a small sample size. This may lead to Type II errors in the identification of risk factors and does not allow for subgroup analyses. While the total number of young people (aged 12–25 years) who were engaged in at least one appointment with *headspace* Launceston in over the previous 12 months was approximately 1100, the exact number of eligible participants (aged 15–25 years) is unknown due to limitations in accessing this data from the *headspace* referral system. However, the current study employed multiple recruitment strategies to reach this at‐risk group, highlighting challenges in sampling this population that should be considered in future research. In addition, the regional location of the study setting further limits the generalisability of the results to other populations and geographical areas. Lastly, the sample contained a high proportion of female respondents (68%) which, while not uncommon in research related to food security,^26^ is a factor that should be considered when interpreting the results of this study. Ongoing research could focus on more opportunistic or face‐to‐face data collection strategies, which were not possible throughout 2020/2021 in Australia due to the COVID‐19 pandemic restrictions. The food security screening tool used was selected to minimise participant burden, but it has been reported to be a less‐sensitive measure of food security,^26^ particularly marginal security which includes anxiety over food sufficiency in addition to running out of food.^3^ Utilising this screening tool may be appropriate for staff in a mental health service setting to identify clients at risk of food insecurity so that appropriate referrals, such as to a dietitian for full assessment, and relevant interventions can be initiated. However, future in‐depth research in this setting should consider utilising a food security tool that can classify respondents according to the severity of food insecurity, or a tool that considers multiple dimensions of food insecurity particularly around diet quality and challenges experienced accessing nutritious foods. In addition, the dietary intake variables were selected based on recommendations from previous research in adolescents that suggested very simple dietary screening questions are preferable to more comprehensive dietary assessment techniques.^27^ Future in‐depth research may consider exploring diet in a more comprehensive way in this population, perhaps through interviewer administered 24‐h recalls or a food frequency questionnaire that could provide a more complete and valid measure of dietary intake.

In conclusion, this research found a high occurrence of food insecurity in young people attending a youth mental health service, with those living independently or in unstable housing, or receiving government financial assistance at the highest risk indicating that financial access to food is a major contributor to food insecurity in this group. Given the results of the study, there is a clear need for additional research in Australia to explore food insecurity in a representative sample of young people, including the main demographic drivers and relationship with mental and physical health outcomes. This should be a priority given the long‐term impacts on physical health^47^ and education outcomes^48^ for young people experiencing food insecurity over their lifespan. Most relevant for this study are the long‐term impacts on mental health, where food insecurity in childhood has been shown to be associated with a higher risk of mental health issues in adolescents and young adults.^49^ Regardless of food security status, this study showed that dietary intake of fruits, vegetables and breakfast was poor, and respondents regularly consumed takeaway foods and sugar‐sweetened beverages. The results of this study could be utilised by managers and funders of youth mental health services to inform changes in service delivery that support improving diet. Literature suggests that routine screening and improved access to interventions by dietitians should be considered given the relationship between mental health, diet quality and food security and that dietitians have unique skills and knowledge to address these issues.^50^, ^51^ Further research surrounding the benefit of integrating dietetic support in the youth mental health setting is warranted. Lastly, continued advocacy for increasing government financial support packages will be paramount in reducing the financial limitations to food access in this at‐risk group.

## AUTHOR CONTRIBUTIONS

Conceptualization, KK, SM, TM, HB; methodology, all authors; formal analysis, KK and DV; investigation, TM, HB; resources, HB; data curation, TM and KK; writing—original draft preparation, KK; writing—review and editing, all authors.; project administration, HB; funding acquisition, HB. All authors have read and agreed to the published version of the manuscript.

## CONFLICT OF INTEREST

The authors declare no conflicts of interest.

## Data Availability

Data utilised in this manuscript are available upon reasonable written request to the corresponding author.

## References

[1] Food and Agriculture Organization, FAO. The State of Food and Agriculture 2001. No. 33. Food & Agriculture Org., 2001.

[2] Australian Bureau of Statistics. Australian Health Survey: Nutrition—State and Territory Results 2011–2012 Cat No. 4364.0. 55.009. (2015).

[3] Seivwright AN , Callis Z , Flatau P . Food insecurity and socioeconomic disadvantage in Australia. Int J Environ Res Public Health. 2020;17:559. 10.3390/ijerph17020559 PMC7014009 31952327

[4] McKay FH , Haines BC , Dunn M . Measuring and understanding food insecurity in Australia: a systematic review. Int J Environ Res Public Health. 2019;16:476. 10.3390/ijerph16030476 PMC6388276 30736305

[5] Godrich SL , Davies CR , Darby J , Devine A . What are the determinants of food security among regional and remote Western Australian children? Aust N Z J Public Health. 2017;41:172‐177. 28110492 10.1111/1753-6405.12636

[6] Russell J , Flood V , Yeatman H , Mitchell P . Prevalence and risk factors of food insecurity among a cohort of older Australians. J Nutr Health Aging. 2013;18:3‐8.10.1007/s12603-013-0339-624402381

[7] Crawford B , Yamazaki R , Franke E , Amanatidis S , Ravulo J , Torvaldsen S . Is something better than nothing? Food insecurity and eating patterns of young people experiencing homelessness. Aust N Z J Public Health. 2015;39:350‐354. 25902911 10.1111/1753-6405.12371

[8] Pourmotabbed A , Moradi S , Babaei A , et al. Food insecurity and mental health: a systematic review and meta‐analysis. Public Health Nutr. 2020;23:1778‐1790. 32174292 10.1017/S136898001900435X PMC10200655

[9] Gundersen C , Ziliak JP . Food insecurity and health outcomes. Health Aff. 2015;34:1830‐1839. 10.1377/hlthaff.2015.0645 26526240

[10] Bhawra J , Kirkpatrick SI , Hammond D . Food insecurity among Canadian youth and young adults: insights from the Canada food study. Can J Public Health. 2021;112:663‐675. 33620691 10.17269/s41997-020-00469-1 PMC8225736

[11] Larson N , Laska MN , Neumark‐Sztainer D . Food insecurity, diet quality, home food availability, and health risk behaviors among emerging adults: findings from the EAT 2010‐2018 study. Am J Public Health. 2020;110:1422‐1428. 32673120 10.2105/AJPH.2020.305783 PMC7427214

[12] Tarasuk V , Cheng J , Gundersen C , de Oliveira C , Kurdyak P . The relation between food insecurity and mental health care service utilization in Ontario. Can J Psychiatry. 2018;63:557‐569. 29307216 10.1177/0706743717752879 PMC6099753

[13] Kirkpatrick SI , Tarasuk V . Food insecurity is associated with nutrient inadequacies among Canadian adults and adolescents. J Nutr. 2008;138:604‐612. 18287374 10.1093/jn/138.3.604

[14] Bhattacharya J , Currie J , Haider S . Poverty, food insecurity, and nutritional outcomes in children and adults. J Health Econ. 2004;23:839‐862. 15587700 10.1016/j.jhealeco.2003.12.008

[15] Paus T , Keshavan M , Giedd JN . Why do many psychiatric disorders emerge during adolescence? Nat Rev Neurosci. 2008;9:947‐957. 19002191 10.1038/nrn2513 PMC2762785

[16] Australian Institute of Health and Welfare . Mental illness [Internet]. Australian Institute of Health and Welfare; 2021 Accessed January 18, 2022. Available from: https://www.aihw.gov.au/reports/children‐youth/mental‐illness

[17] Wattick RA , Hagedorn RL , Olfert MD . Relationship between diet and mental health in a young adult Appalachian college population. Nutrients. 2018;10:957. 10.3390/nu10080957 PMC6115820 30044399

[18] O'Neil A , Quirk SE , Housden S , et al. Relationship between diet and mental health in children and adolescents: a systematic review. Am J Public Health. 2014;104:e31‐e42. 10.2105/AJPH.2014.302110 PMC4167107 25208008

[19] Burke MP , Martini LH , Çayır E , Hartline‐Grafton HL , Meade RL . Severity of household food insecurity is positively associated with mental disorders among children and adolescents in the United States. J Nutr. 2016;146:2019‐2026. 27581581 10.3945/jn.116.232298

[20] Shankar P , Chung R , Frank DA . Association of food insecurity with children's behavioral, emotional, and academic outcomes: a systematic review. J Dev Behav Pediatr. 2017;38:135‐150. 28134627 10.1097/DBP.0000000000000383

[21] Australian Bureau of Statistics, ABS . National, State and Territory Population. ABS; 2021 Accessed January 18, 2022. Available from: https://www.abs.gov.au/statistics/people/population/national‐state‐and‐territory‐population/latest‐release#key‐statistics

[22] Australian Bureau of Statistics, ABS . Tasmania 2016 census QuickStats ABS. ABS; 2016 Accessed January 18, 2022. Available from: https://quickstats.censusdata.abs.gov.au/census_services/getproduct/census/2016/quickstat/6?opendocument#:~:text=People%20%E2%80%94%20demographics%20%26%20education&text=In%20the%202016%20Census%2C%20there,up%204.6%25%20of%20the%20population.&text=The%20median%20age%20of%20people%20in%20Tasmania%20was%2042%20years.

[23] Australian Government Department of Health . (n.d.). Health Workforce Locater. Australian Government Department of Health; Accessed January 18, 2022. Available from: https://www.health.gov.au/resources/apps‐and‐tools/health‐workforce‐locator/health‐workforce‐locator.

[24] The Department of Premier and Cabinet (DPAC) of the Tasmanian State Government . 2021. Mental Health Referral Guide for Young People in Tasmania. DPAC; Accessed January 18, 2022. Available from: https://www.dpac.tas.gov.au/__data/assets/pdf_file/0005/237461/Mental_Health_Referral_Guide_for_Young_People_in_Tasmania_A3_Poster.pdf.

[25] Bridgman H , Mawer T , McGowan C , et al. Cornerstone Youth Services Nutrition and Physical Activity Capacity Building Project. University of Tasmania; 2021.

[26] McKechnie R , Turrell G , Giskes K , Gallegos D . Single‐item measure of food insecurity used in the National Health Survey may underestimate prevalence in Australia. Aust N Z J Public Health. 2018;42:389‐395. 30035843 10.1111/1753-6405.12812

[27] Flood VM , Webb KL , Rangan AM . Recommendations for short questions to assess food consumption in children for the NSW Health Surveys. 2005.

[28] Australian Government Department of Health . Modified Monash Model. Australian Government Department of Health; 2019.

[29] Nolan M , Williams M , Rikard‐Bell G , Mohsin M . Food insecurity in three socially disadvantaged localities in Sydney, Australia. Health Promot J Austr. 2006;17:247‐254. 17176242 10.1071/he06247

[30] Murray S , Peterson C , Primo C , et al. Prevalence of food insecurity and satisfaction with on‐campus food choices among Australian university students. Int J Sustain High Educ. 2021;22:731‐746.

[31] Teasdale SB , Morell R , Lappin JM , Curtis J , Watkins A , Ward PB . Prevalence and correlates of food insecurity in community‐based individuals with severe mental illness receiving long‐acting injectable antipsychotic treatment. Br J Nutr. 2020;124:470‐477. 32234106 10.1017/S0007114520001191

[32] Foodbank . 2021. Foodbank Hunger Report 2021, Foodbank, Accessed January 18, 2022. Available from: https://reports.foodbank.org.au/wp‐content/uploads/2021/10/2021‐Foodbank‐Hunger‐Report‐PDF.pdf.

[33] Temple JB , Booth S , Pollard CM . Social assistance payments and food insecurity inAustralia: evidence from the HouseholdExpenditure survey. Int J Environ Res Public Health. 2019;16:455. 10.3390/ijerph16030455 PMC6388211 30720768

[34] Australian Parliment. Characteristics and Use of Casual Employees in Australia. Australian Parliment; 2022. Accessed January 11, 2022. Available from: https://www.aph.gov.au/About_Parliament/Parliamentary_Departments/Parliamentary_Library/pubs/rp/rp1718/CasualEmployeesAustralia.

[35] Australian Institute of Health and Welfare. (2021). Housing Affordability. [Internet]: Australian Institute of Health and Welfare, January 18, 2022. Available from: https://www.aihw.gov.au/reports/australias‐welfare/housing‐affordability.

[36] Daley J , Wood D , Coates B , et al. The Recovery Book: What Australian Governments Should Do Now. Grattan Institute; 2020.

[37] Australia's Mental Health Think Tank . COVID‐19 and Australia's Mental Health: An Overview of Academic Literature, Policy Documents, Lived Experience Accounts and Community Reports. Australia's Mental Health Think Tank; 2021 January 18, 2022. Available from: https://apo.org.au/node/314410

[38] Subramaniam M , Zhang Y , Shahwan S , et al. Employment of young people with mental health conditions: making it work. Disabil Rehabil. 2020;44(1‐11):2033‐2043. Employment of young people with mental health conditions: makin. 10.1080/09638288.2020.1822932 32988237

[39] Wetherill MS , White KC , Rivera C . Food insecurity and the nutrition care process: practical applications for dietetics practitioners. J Acad Nutr Diet. 2018;118(12):2223‐2234. doi:10.1016/j.jand.2017.08.114 29217123 PMC5986582

[40] Teasdale SB , Ward PB , Rosenbaum S , Samaras K , Stubbs B . Solving a weighty problem: systematic review and meta‐analysis of nutrition interventions in severe mental illness. Br J Psychiatry. 2017;210:110‐118. 27810893 10.1192/bjp.bp.115.177139

[41] Wise J . Dietitians have important role in mental health team, say researchers. Br Med J (Online). 2016;355:i5913. 10.1136/bmj.i5913 27811007

[42] Australian Institute of Health and Welfare . Nutrition. [Internet]: Australian Institute of Health and Welfare, 2021. Accessed April 28, 2022. Available from: https://www.aihw.gov.au/reports/children‐youth/nutrition

[43] Teasdale SB , Burrows TL , Hayes T , et al. Dietary intake, food addiction and nutrition knowledge in young people with mental illness. Nutr Diet. 2020;77:315‐322. 31243895 10.1111/1747-0080.12550

[44] Mullan B , Wong C , Kothe E , O'Moore K , Pickles K , Sainsbury K . An examination of the demographic predictors of adolescent breakfast consumption, content, and context. BMC Public Health. 2014;14:264. 24645936 10.1186/1471-2458-14-264 PMC4000053

[45] Pepetone A , Vanderlee L , White CM , Hammond D , Kirkpatrick SI . Food insecurity, food skills, health literacy and food preparation activities among young Canadian adults: a cross‐sectional analysis. Public Health Nutr. 2021;24:2377‐2387. 33648617 10.1017/S1368980021000719 PMC10195398

[46] Chi DL , Masterson EE , Carle AC , Mancl LA , Coldwell SE . Socioeconomic status, food security, and dental caries in US children: mediation analyses of data from the National Health and nutrition examination survey, 2007–2008. Am J Public Health. 2014;104:860‐864. 24625141 10.2105/AJPH.2013.301699 PMC3987603

[47] Tamiru D , Belachew T . The association of food insecurity and school absenteeism: systematic review. Agriculture & Food Security. 2017;6:5.

[48] McIntyre L , Williams JV , Lavorato DH , Patten S . Depression and suicide ideation in late adolescence and early adulthood are an outcome of child hunger. J Affective Disorders. 2013;150:123‐129. 10.1016/j.jad.2012.11.029 23276702

[49] Furness T , Wallace E , McElhinney J , McKenna B , Cuzzillo C , Foster K . Colocating an accredited practising dietitian to an adult community mental health service: an exploratory study. Int J Ment Health Nurs 2018;27(6):1709‐1718. doi:10.1111/inm.12470 29704288

[50] Teasdale SB , Latimer G , Byron A , et al. Expanding collaborative care: integrating the role of dietitians and nutrition interventions in services for people with mental illness. Australas Psychiatry. 2018 Feb;26(1):47‐49. 28869391 10.1177/1039856217726690

[51] National Health and Medical Research Council. Australian Dietary Guidelines: Eat for Health. 2013; National Health and Medical Research Council.

